# Prenatal exposure to TiO_2_ nanoparticles in mice causes behavioral deficits with relevance to autism spectrum disorder and beyond

**DOI:** 10.1038/s41398-018-0251-2

**Published:** 2018-09-20

**Authors:** Tina Notter, Leonie Aengenheister, Ulrike Weber-Stadlbauer, Hanspeter Naegeli, Peter Wick, Urs Meyer, Tina Buerki-Thurnherr

**Affiliations:** 1 Institute of Pharmacology and Toxicology, University of Zurich-Vetsuisse, Winterthurerstrasse 260, 8057 Zurich, Switzerland; 2 Neuroscience Center Zurich, University of Zurich and ETH Zurich, Winterthurerstrasse 190, 8057 Zurich, Switzerland; 3 Particles-Biology Interactions, Empa, Swiss Federal Laboratories for Materials Science and Technology, Lerchenfeldstrasse 5, 9014 St. Gallen, Switzerland

## Abstract

Environmental factors are involved in the etiology of autism spectrum disorder (ASD) and may contribute to the raise in its incidence rate. It is currently unknown whether the increasing use of nanoparticles such as titanium dioxide (TiO_2_ NPs) in consumer products and biomedical applications may play a role in these associations. While nano-sized TiO_2_ is generally regarded as safe and non-toxic, excessive exposure to TiO_2_ NPs may be associated with negative health consequences especially when occurring during sensitive developmental periods. To test if prenatal exposure to TiO_2_ NPs alters fetal development and behavioral functions relevant to ASD, C57Bl6/N dams were subjected to a single intravenous injection of a low (100 µg) or high (1000 µg) dose of TiO_2_ NPs or vehicle solution on gestation day 9. ASD-related behavioral functions were assessed in the offspring using paradigms that index murine versions of ASD symptoms. Maternal exposure to TiO_2_ NPs led to subtle and dose-dependent impairments in neonatal vocal communication and juvenile sociability, as well as a dose-dependent increase in prepulse inhibition of the acoustic startle reflex of both sexes. These behavioral alterations emerged in the absence of pregnancy complications. Prenatal exposure to TiO_2_ NPs did not cause overt fetal malformations or changes in pregnancy outcomes, nor did it affect postnatal growth of the offspring. Taken together, our study provides a first set of preliminary data suggesting that prenatal exposure to nano-sized TiO_2_ can induce behavioral deficits relevant to ASD and related neurodevelopmental disorders without inducing major changes in physiological development. If extended further, our preclinical findings may provide an incentive for epidemiological studies examining the role of prenatal TiO_2_ NPs exposure in the etiology of ASD and other neurodevelopmental disorders.

## Introduction

Autism spectrum disorder (ASD) is a group of neurodevelopmental disorders with increasing incidence rates. According to the Center for Disease Control and Prevention, the prevalence of ASD has doubled within the last decade^1^, with current estimates suggesting that 1 in 68 American children is diagnosed with ASD^1^. Whilst increased awareness, reclassification, diagnostic expansion, and inclusion of milder neurodevelopmental deficits likely contribute to the marked increase in the rate of diagnosed ASD^2^, changes in the environment may add to this increase as well^3,4^. The latter hypothesis is supported by recent studies suggesting that 40–50% of variance in ASD liability may be determined by environmental risk factors^5,6^.

In accordance with the developmental origin of ASD, most of the identified environmental risk factors act during pre- or perinatal periods. Examples of pre- or perinatal risk factors include maternal exposure to infection and/or inflammation^7–10^, maternal allergic-asthma^11,12^, maternal autoantibodies reacting with fetal proteins^13,14^, obstetric complications such as maternal hypertension and preeclampsia^15,16^, and pre- and/or perinatal exposure to (traffic-related) air pollution^17,18^. The latter is of particular interest as it may be related to, or even driven by, the continuing urbanization, which in itself is considered an environmental risk factor of ASD^19^. In addition to air pollution, early-life exposure to various other environmental toxicants, including mercury, lead, arsenic, polychlorinated biphenyls and toluene, are known causes of neurodevelopmental disorders and may play a role in the etiology of ASD^20^.

The increasing use of nanoparticles (NPs) in various consumer products and biomedical applications may represent another challenge for public health^21,22^. NPs are particles with at least one dimension below 100 nm and can be engineered with distinctive compositions, sizes, shapes, and surface chemistries^23^. Among the most frequently produced and used NPs are those that are based on titanium dioxide (TiO_2_)^24,25^. Their unique physicochemical and optical properties enable the application of TiO_2_ NPs as additives in various consumer products, including toothpaste, sunscreen, paints, and food (E171)^24,26^. In 2005 the worldwide production of titanium powder was approximately 5 million tons^27^. According to recent estimations, the proportion of nano-sized TiO_2_ in titanium powder increased from 2.5% in 2009 to 10% in 2015^27,28^.

Nano-sized particles such as TiO_2_ NPs can either penetrate intact cell membranes^29^ or can be taken up via endocytosis to cross biological barriers^30,31^. Hence, even if the majority of TiO_2_ NPs may be excreted rapidly upon uptake, a certain amount of nano-sized TiO_2_ particles can be absorbed and disseminated into various tissues by the systemic circulation^26,32,33^. While TiO_2_ NPs are generally regarded as safe and non-toxic, a number of studies have raised possible health concerns in relation to the increasing use of nano-sized TiO_2_ particles^26,34,35^. Exposure to TiO_2_ NPs may be associated with negative health consequences especially when occurring during developmental periods. For example, studies in mice have found that TiO_2_ NPs can cross the placental–fetal barrier and induce moderate to severe malformations of the developing fetuses^36,37^. Prenatal exposure to TiO_2_ NPs in mice was further shown to alter gene expression profiles in the developing brain^38^ and to change cortical neurotransmitter levels in adolescent offspring^39^. Moreover, recent studies suggest that prenatal TiO_2_ NP exposure can induce long-term behavioral deficits relevant to depression^40^ and cognitive impairments in rats^41,42^.

In keeping with the increasing use of TiO_2_ NPs and the rise in the incidence rates of ASD, we sought to evaluate whether prenatal exposure to nano-sized TiO_2_ may alter brain and behavioral development in a manner that is relevant to ASD. To this end, we exposed pregnant mice to distinct doses of TiO_2_ NPs or control treatment and explored ASD-related behavioral functions in the resulting offspring. The behavioral tests included paradigms assessing murine versions of core symptoms of ASD, including impairments in social interaction, deficits in verbal communication, and presence of stereotyped/repetitive behaviors^43^. We also included tests for anxiety-like behavior and prepulse inhibition in attempts to examine ASD-related symptoms of anxiety and altered sensorimotor gating^44,45^. To assess the distribution of TiO_2_ NPs following maternal exposure, we measured the contents of titanium (Ti) in distinct maternal tissues (liver, spleen, plasma), placenta, fetal liver, and fetal brain. All investigations were conducted in male and female offspring in order to reveal potential sex-dependent effects.

## Material and methods

### Animals

C57Bl6/N mice were used throughout this study (Supplementary Information). All procedures involving animal experimentation had been previously approved by the Cantonal Veterinarian’s Office of Zurich, and all efforts were made to minimize the number of animals used and their suffering.

### TiO_2_ NP suspension

TiO_2_ anatase NPs (NM-101) were provided by the Joint Research Center (JRC, Ispra, Italy). The TiO_2_ NP powder was dissolved in sterile and pyrogen-free phosphate-buffered saline (PBS) (D8537, Sigma-Aldrich, Switzerland) (5 mg/mL) via probe sonication (5 min at 13 W, on ice; Branson sonifier 250, Branson Ultrasonic Co., Danbury, CT, USA). Detailed information about the characterization of TiO_2_ NP suspension is provided in the Supplementary Information.

### Prenatal TiO_2_ NP exposure

C57BL6/N female mice were subjected to a timed mating procedure as described previously^46^. Pregnant dams were subjected to a single intravenous injection of the 100 µg or 1000 µg TiO_2_ NP solution (see above) or vehicle solution (PBS) on gestation day 9 (GD) 9 (Supplementary Information). Two cohorts of pregnant mice were generated under identical experimental and housing conditions (see Supplementary Information). The first cohort of dams was designated to fetal developmental and Ti tissue distribution studies and the second cohort was used to generate offspring for behavioral studies (see Supplementary Information).

The methods used for maternal and fetal tissue collection for ICP-MS and evaluation of fetal development are described in the Supplementary Information.

### Behavioral testing in the offspring

Male and female offspring were behaviorally tested in paradigms that indexed murine versions of ASD symptoms, including deficits in verbal communication, impairments in social interaction, and presence of stereotyped/repetitive behaviors^43^. Behavioral assessing also included tests for anxiety-like behavior and prepulse inhibition in attempts to examine ASD-related symptoms of anxiety and altered sensorimotor gating^44,45^. While verbal communication was assessed in neonatal mice on postnatal day (PND) 6, all other tests were conducted in a separate subgroup of offspring when they reached the juvenile stage (i.e., between PND 28 and 42). Juvenile offspring were repeatedly tested in the following order, with a testing-free resting period of 2 days between individual tests: (1) open field test, (2) social interaction test, (3) self-grooming test, and (4) prepulse inhibition (PPI) test. A detailed description of the test apparatuses and procedures is provided in the Supplementary Information.

### Statistical analyses

All data met the assumptions of normal distribution and equality of variance. All data were analyzed using parametric analysis of variance (ANOVA) as described in the Supplementary Information. Whenever appropriate, all ANOVAs were followed by Fisher’s least significant difference (LSD) post-hoc tests. All statistical analyses were performed using StatView (version 5.0; Abacus, Phoenix, AZ, USA) implemented on a PC running the Windows XP operating system, and Prism software (version 7.0; GraphPad Software, La Jolla, CA, USA). Statistical significance was set at *p* < 0.05 for all tests. No exclusion criteria were applied.

## Results

### Tissue distribution of TiO_2_ NPs

Well-characterized anatase TiO_2_ NPs from the JRC repository (NM-101) were used for this study (see Supplementary Table 2). Ultrasensitive sector field-inductively coupled plasma mass spectrometry revealed that maternal TiO_2_ NP treatment led to a dose- and tissue-dependent accumulation of Ti, as supported by the significant main effect of prenatal treatment (*F*_(2, 72)_ = 110.5, *p* < 0.001), tissue compartment (*F*_(5, 72)_ = 62.36, *p* < 0.001), and their interaction (*F*_(10,72)_ = 44.6, *p* < 0.001). As illustrated in Supplementary Fig. 1, Ti was found to accumulate in maternal liver and spleen. Post-hoc comparisons of maternal liver and spleen samples confirmed a dose-dependent increase in Ti between control animals (0 µg) and animals exposed to 100 µg (liver: *p* < 0.001, spleen: *p* < 0.001) or 1000 µg (liver: *p* < 0.001, spleen: *p* < 0.001), and between animals exposed to 100 µg and 1000 µg (liver: *p* < 0.001, spleen: *p* < 0.001). On the other hand, there was no significant accumulation of Ti in maternal plasma, placenta, fetal liver, and fetal brain (Supplementary Fig. 1).

### Effects of maternal TiO_2_ NP exposure on pregnancy outcomes and offspring development

Maternal exposure to TiO_2_ NPs did not affect fetal length (*F*_(2,21)_ = 0.129, *p* = 0.879), fetal weights (*F*_(2,21)_ = 0.845, *p* = 0.444), and fetal brain weights (*F*_(2,21)_ = 0.036, *p* = 0.964) (Supplementary Fig. 2b). Likewise, a gross examination of the fetal morphology did not reveal noticeable differences between fetuses of control dams and dams exposed to TiO_2_ NPs (Supplementary Fig. 2a). Hence, maternal exposure to TiO_2_ NPs did not cause overt signs of fetal malformations.

In agreement with these findings, maternal exposure to TiO_2_ NPs did not affect litter sizes (*F*_(2,22)_ = 0.270, *p* = 0.766; Supplementary Fig. 2c) or the male/female ratio of the delivered pups (*F*_(2,22)_ = 1.63, *p* = 0.23; Supplementary Fig. 2d). The offspring’s body weights were also not changed by the prenatal manipulation. As expected, the offspring’s body weights increased from neonatal (PND 6) to juvenile (PND 21 and 28) ages (main effect of age: *F*_(2,149)_ = 2430.884, *p* < 0.001; Supplementary Fig 2e), and this age effect was not influenced by prenatal treatment (main effect of prenatal treatment: *F*_(2,149)_ = 0.046, *p* = 0.955; interaction between prenatal treatment and age: *F*_(4,149)_ = 0.394, *p* = 0.813). Female offspring generally weighed less than male offspring (main effect of sex: *F*_(1,149)_ = 65.507, *p* < 0.001), regardless of their prenatal treatment conditions (interaction between prenatal treatment and sex: *F*_(2,149)_ = 1.093, *p* = 0.338). Together, these findings indicate that maternal exposure to TiO_2_ NPs did not affect pregnancy outcomes or postnatal development of the offspring.

### Effects of maternal TiO_2_ NP exposure on neonatal ultrasonic vocalization

**Fig. 1:**
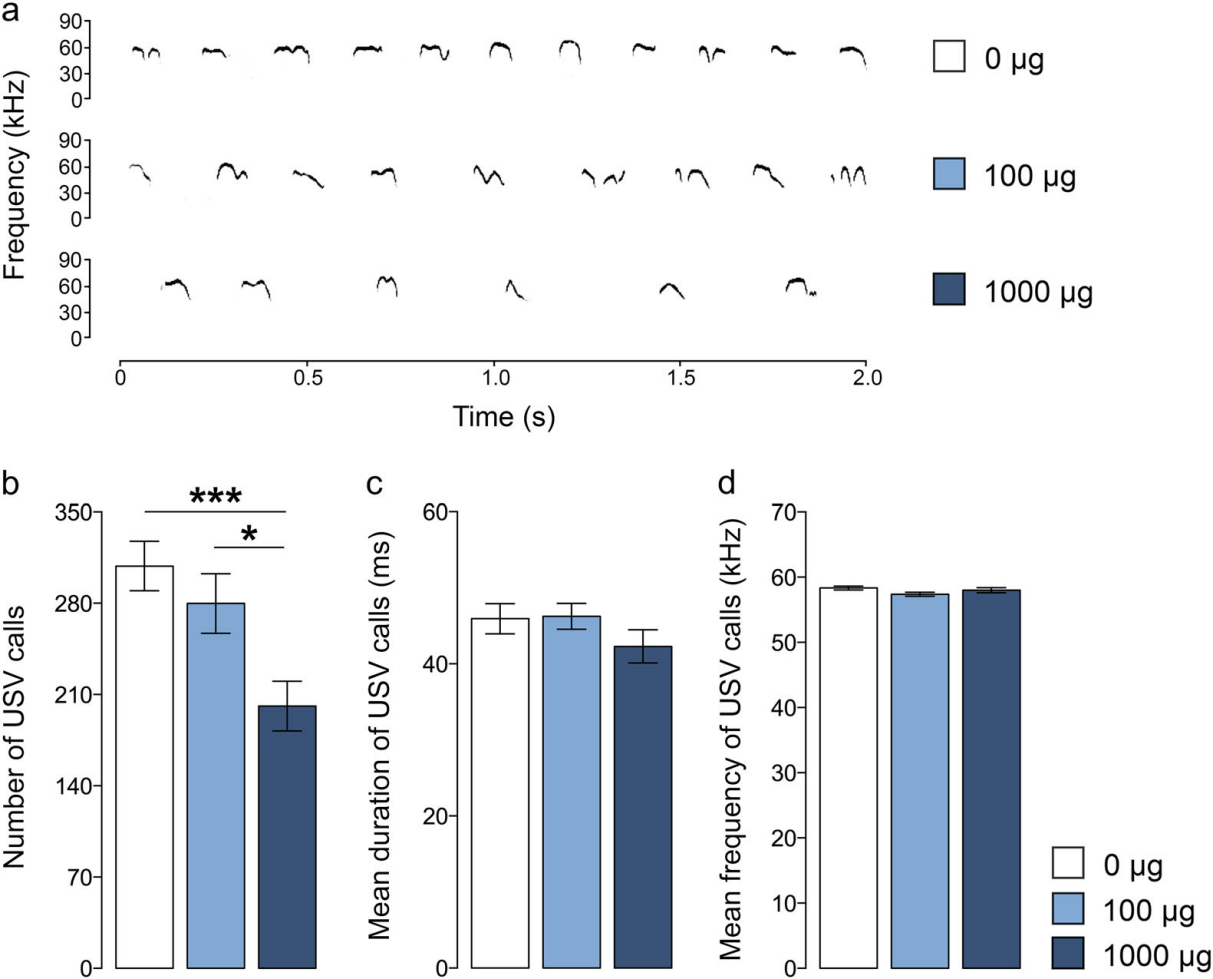
Ultrasonic vocalizations (USV) measured in neonatal offspring prenatally exposed to 0 (=vehicle), 100, or 1000 µg TiO_2_. **a** Representative spectrograms of USVs emitted from separated pups. **b** The bar plot represents the number of emitted USV calls. *p < 0.05 and ***p < 0.001. **c** The bar plot depicts the mean duration (ms) of emitted USV calls. **d** The bar plot depicts the mean dominant frequency of emitted USV calls in kHz. All data are based on *N*(0 µg) = 16 (8m, 8f), *N*(100 µg) = 16 (8m, 8f) and *N*(1000 µg) = 17 (9m, 8f). All values are means ± SEM

Verbal communication was assessed in neonatal mice by measuring USV calls upon acute separation from their littermates and rearing mothers. Prenatal TiO_2_ NP exposure led to a dose-dependent decrease in the total numbers of USV calls emitted from pups that were separated from their mothers (main effect of prenatal treatment: *F*_(2,43)_ = 5.361, *p* < 0.01) (Fig. 1b). Post-hoc analyses confirmed significantly decreased USV calls in neonates of mothers that were treated with 1000 µg TiO_2_ NPs relative to control neonates (*p* < 0.01) or to neonates of mothers that were treated with 100 µg TiO_2_ NPs (*p* < 0.05) (Fig. 1b). These effects were sex-independent (main effect of sex: *F*_(1,43)_ = 1.703, *p* = 0.199; interaction between prenatal treatment and sex: *F*_(2,43)_ = 0.028, *p* = 0.973). In contrast to its effects on the number of USVs, prenatal TiO_2_ NP exposure did not affect the mean duration of USV calls (main effect of prenatal treatment: *F*_(2,43)_ = 1.187, *p* = 1.187; interaction between prenatal treatment and sex: *F*_(2,43)_ = 0.035, *p* = 0.966) (Fig. 1c). There were also no group differences in terms of the mean dominant frequency of emitted USV calls (main effect of prenatal treatment: *F*_(2,43)_ = 2.534, *p* = 0.091; interaction between prenatal treatment and sex: *F*_(2,43)_ = 1.437, *p* = 0.249) (Fig. 1d).

### Effects of maternal TiO_2_ NP exposure on sociability and repetitive behavior

**Fig. 2:**
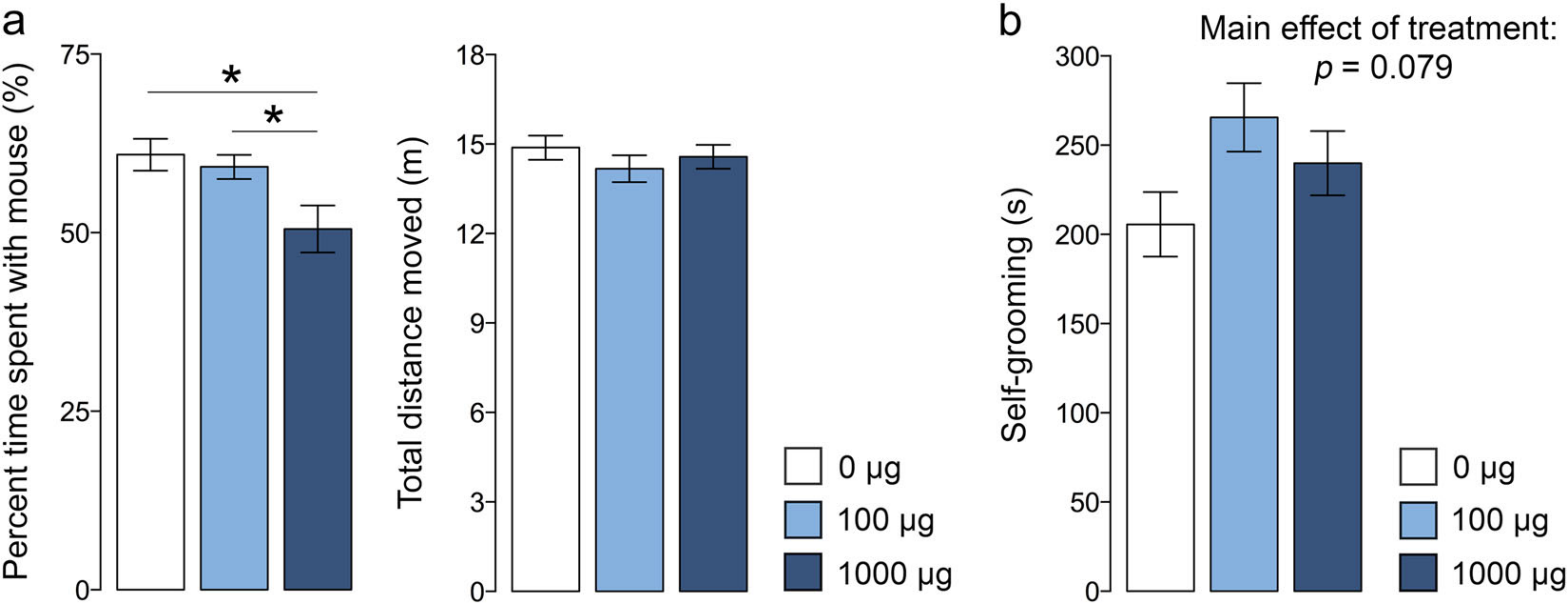
Social interaction and stereotyped/repetitive behavior in juvenile offspring prenatally exposed to 0 (=vehicle), 100, or 1000 µg TiO_2_. **a** The bar plots depict the percent time spent with an unfamiliar mouse and the total distance moved during the social interaction test. *p < 0.05. **b** The bar plot depicts the time spent self-grooming during a period of 10 min. All data are based on *N*(0 µg) = 20 (10m, 10f), *N*(100 µg) = 19 (10m, 9f), *N*(1000 µg) = 20 (10m, 10f). All values are means ± SEM

Sociability of juvenile offspring was assessed using a modified version of the three-chamber social interaction test^46,47^. In this test, sociability was indexed as the relative exploration time between an unfamiliar, congenic mouse of the same sex and an inanimate dummy object. Prenatal TiO_2_ NP exposure caused a dose-dependent deficit in social approach behavior (Fig. 2a), as supported by the main effect of prenatal treatment (*F*_(2,53)_ = 4.838, *p* < 0.05) and by the subsequent post-hoc analyses revealing a significant difference between offspring of mothers that were treated with 1000 µg TiO_2_ NPs and control offspring (*p* < 0.05) or offspring of mothers that were treated with 100 µg TiO_2_ NPs (*p* < 0.05). The effect of prenatal TiO_2_ NP exposure on sociability emerged independently of sex (main effect of sex: *F*_(1,53)_ = 0.959, *p* = 0.332; interaction between prenatal treatment and sex: *F*_(2,53)_ = 0.091, *p* = 0.914) and was not associated with concomitant changes in locomotor activity (Fig. 2a). The latter was indexed by the total distance moved during the social interaction test, which did not yield any significant effects (main effect of prenatal treatment: *F*_(2,53)_ = 0.771, *p* = 0.468; main effect of sex: *F*_(1,53)_ = 2.740, *p* = 0.104; interaction between prenatal treatment and sex: *F*_(2,53)_ = 0.247, *p* = 0.782).

A self-grooming test was used to explore repetitive behavior in juvenile offspring. As illustrated in Fig. 2b, offspring of mothers that were treated with TiO_2_ NPs showed a trend towards increased levels of self-grooming relative to control offspring (main effect of prenatal treatment: *F*_(2,53)_ = 2.668, *p* = 0.079). The main effect of sex (*F*_(1,53)_ = 0.924, *p* = 0.341) and its interaction with prenatal treatment (*F*_(2,53)_ = 0.772, *p* = 0.462) were far from being significant.

### Effects of maternal TiO_2_ NP exposure on sensorimotor gating

**Fig. 3:**
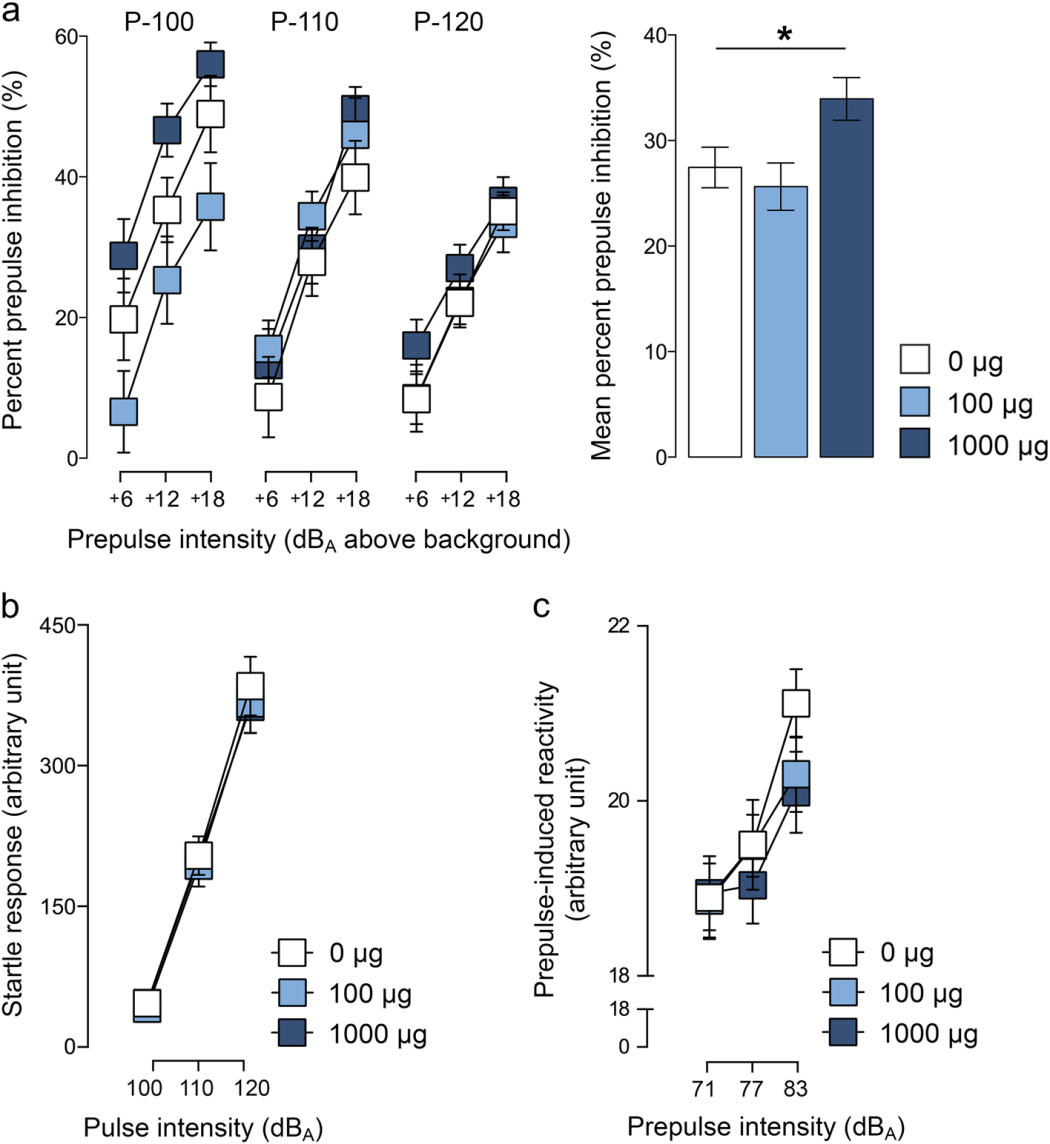
Prepulse inhibition of the acoustic startle reflex in juvenile offspring prenatally exposed to 0 (=vehicle), 100, or 1000 µg TiO_2_. **a** The line plot depicts percent prepulse inhibition as a function of different pulse intensities (P-100, P-110 and P-120, corresponding to 110, 110, and 120 dB_A_) and prepulse intensities (+6, +12, and +18 dB_A_ above background of 65 dB_A_). The bar plot shows the mean prepulse inhibition scores across all pulse and prepulse conditions. *p *<* 0.05. **b** The line plot depicts the startle response to pulse-alone stimuli as a function of pulse intensities (110, 110, and 120 dB_A_). **c** The line plot depicts the reactivity to prepulse-alone stimuli as a function of prepulse intensities (71, 77, and 83 dB_A_). All data are based on *N*(0 µg) = 20 (10m, 10f), *N*(100 µg) = 19 (10m, 9f), *N*(1000 µg) = 20 (10m, 10f). All values are means ± SEM

Sensorimotor gating was assessed in juvenile offspring using PPI of the acoustic startle reflex. Prenatal TiO_2_ NP exposure significantly altered % PPI, as indicated by the main effect of prenatal treatment (*F*_(2,53)_ = 4.469, *p* < 0.05). Post-hoc analyses revealed that offspring prenatally exposed to 1000 µg TiO_2_ NPs displayed significantly increased % PPI relative to offspring exposed to 100 µg TiO_2_ NPs (*p* < 0.01) and control offspring (*p* < 0.05) (Fig. 3a). The effect of prenatal TiO_2_ NP exposure on % PPI was independent of sex (main effect of sex: *F*_(1,53)_ = 1.710, *p* = 0.197; interaction with prenatal treatment and sex: *F*_(2,53)_ = 0.260, *p* = 0.772). The increase in % PPI displayed in offspring of mothers that were exposed to 1000 µg TiO_2_ NPs appeared to be largest in conditions, in which 100 dB_A_ stimuli served as pulse stimuli (Fig. 3a); however, the interaction between prenatal treatment and pulse stimulus was not significant (*F*_(4,106)_ = 2.187, *p* = 0.085). The interaction between prenatal treatment and prepulse was far from being significant (*F*_(4,106)_ = 0.209, *p* = 0.933), indicating that the effect of prenatal TiO_2_ NP exposure on % PPI emerged independently of prepulse intensity.

Prenatal TiO_2_ NP exposure did not affect the reactivity to pulse-alone trials (*F*_(2,53)_ = 0.161, *p* = 0.852); (Fig. 3b) or prepulse-alone trials (*F*_(2,53)_ = 0.477, *p* = 0.623); (Fig. 3c). As expected^47^, the reactivity to pulse-alone and prepulse-alone trials increased with increasing pulse and prepulse intensities, respectively (main effect of pulse: *F*_(2,106)_ = 378.790, *p* < 0.001; main effect of prepulse: *F*_(2,106)_ = 17.886, *p* < 0.001).

### Effects of maternal TiO_2_ NP exposure on anxiety-like behavior

**Fig. 4:**
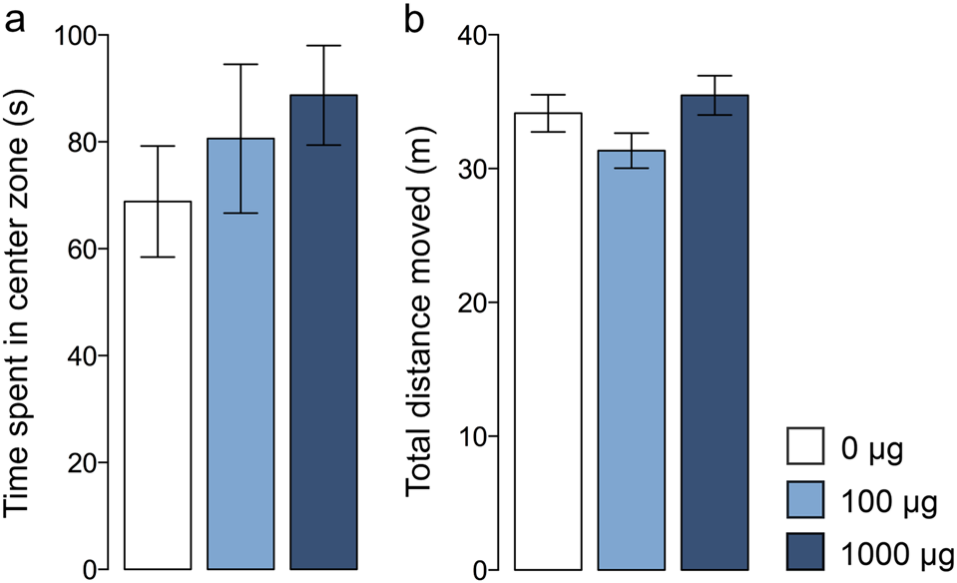
Innate anxiety-like behavior measured in juvenile offspring prenatally exposed to 0 (=vehicle), 100, or 1000 µg TiO_2_. Innate anxiety-like behavior was assessed using a 10 min open field test. **a** The bar plot represents the time spent in the center zone of the open field arena. **b** The bar plot depicts the total distance moved in the entire arena. All data are based on *N*(0 µg) = 20 (10m, 10f), *N*(100 µg) = 19 (10m, 9f), and *N*(1000 µg) = 20 (10m, 10f). All values are means ± SEM

Innate anxiety-like behavior was assessed in juvenile offspring using the open-field exploration task. As illustrated in Fig. 4a, prenatal TiO_2_ NP exposure did not affect the time spent in the center zone of the open field during 10 min of free exploration (main effect of prenatal treatment: *F*_(2,53)_ = 0.773, *p* = 0.467; main effect of sex: *F*_(1,53)_ = 1.167, *p* = 0.285; interaction between prenatal treatment and sex: *F*_(2,53)_ = 0.349, *p* = 0.707). Likewise, the prenatal manipulation did not influence general locomotor activity indexed by the total distance moved (main effect of prenatal treatment: *F*_(2,53)_ = 2.800, *p* = 0.070) (Fig. 4b). As expected^48^, the total distance moved was generally higher in female as compared to male offspring (main effect of sex: *F*_(1,53)_ = 13.76, *p* < 0.001), and this effect similarly emerged in all prenatal treatment groups (interaction between prenatal treatment and sex: *F*_(2,53)_ = 1.976, *p* = 0.149).

## Discussion

The present study demonstrates that maternal exposure to nano-sized TiO_2_ leads to a dose-dependent disruption of behavioral functions in mice. The spectrum of behavioral deficits induced by prenatal TiO_2_ NP exposure included impairments in neonatal vocal communication and juvenile social interaction, as well as increased PPI of the acoustic startle reflex in juvenile offspring. The neonatal deficits in vocal communication were manifest as a reduction in USV rates when pups were separated from their littermates and rearing mother and thus likely reflect altered affective states promoting separation-induced vocal responses. The dose-dependent deficit in social approach behavior induced by prenatal TiO_2_ NP exposure was not associated with concomitant alterations in innate anxiety-like behavior, and therefore, it likely represents a genuine impairment in sociability towards unfamiliar conspecifics. Finally, the observed increase in PPI of the acoustic startle reflex may reflect hypersensitivity to sensory information. The consensus is that PPI reflects the ability to filter out irrelevant information in the early stages of processing so that attention can be directed to more salient environmental features^49^. Generally, stronger and/or more salient prepulses induce higher levels of PPI as they are more efficient in inhibiting the processing of the subsequent pulse stimulus. On speculative grounds, prepulses may be more salient for offspring that were prenatally exposed to the highest dose of TiO_2_ as compared to control offspring, and consequently, they would be more efficacious in inhibiting the subsequent pulse-stimulus processing in TiO_2_-exposed offspring, thereby leading to increased PPI.

Interestingly, the TiO_2_-induced disruption of behavioral functions was independent of the offspring’s sex, indicating similar vulnerabilities of the male and female sex to this environmental hazard. Prenatal exposure to nano-sized TiO_2_ did also not cause overt fetal malformations or changes in pregnancy outcomes, nor did it affect postnatal growth of the offspring. Taken together, our mouse model suggests that prenatal TiO_2_ exposure can induce behavioral abnormalities in both sexes without inducing major changes in physiological development.

### Relevance to autism spectrum disorder and beyond

A number of behavioral alterations emerging in offspring that were prenatally exposed to the highest dose of nano-sized TiO_2_, including impairments in vocal communication and social interaction, resemble core features of ASD^50^. These core deficits often emerge as early as in the first years of life and tend to persist throughout life in ASD subjects^51,52^. It should be noted, however, that our study does not offer a direct link between these two behavioral impairments, given that vocal communication was measured in neonatal offspring, whereas social interaction was assessed at the juvenile stage of life. In addition to vocal and social deficits, the TiO_2_-induced changes in PPI may also have some relevance for ASD. While PPI was found to be decreased in a subset of adults with ASD^53,54^, increased PPI of the acoustic startle reflex has been reported for children with ASD^55^. Our findings recapitulate the latter, given that our study examined PPI in juvenile offspring of TiO_2_-exposed mothers and controls. Increased PPI in children with ASD has been related to hypersensitivity to sensory information, which may stem from changes in the perceived salience of seemingly irrelevant stimuli such as prepulses of low intensity.

Despite the fact that prenatal TiO_2_ exposure resulted in ASD-related behavioral deficits, it should be noted that these deficits were relatively subtle and did not extend to other core behavioral abnormalities that are frequently observed in ASD, including repetitive and anxiety-like behaviors^43^. Furthermore, our findings do by no means imply that prenatal TiO_2_ exposure may cause behavioral abnormalities that are specific to ASD. In fact, this environmental hazard may also contribute to other brain disorders, including depression-like behavior and cognitive deficits. For example, a previous study in rats showed that prenatal exposure to TiO_2_ caused anhedonic behavior and behavioral despair, as assessed using the sucrose preference test and forced swimming test, respectively^40^. Furthermore, maternal administration of TiO_2_ in rats was found to impair various cognitive functions, including spatial and non-spatial learning and memory^41,42^. Since our study did not assess functions in these behavioral and cognitive domains, we cannot directly compare our data to these previous findings. Taken together, however, the available data suggest that prenatal exposure to TiO_2_ may be a general vulnerability factor for various brain disorders with neurodevelopmental etiologies.

### Tissue distribution of TiO_2_ NPs and possible mediating mechanisms

In line with previous systemic exposure studies in rodents^33,56,57^, we found that Ti accumulated primarily in the maternal liver and spleen after i.v. administration of TiO_2_ NPs. By contrast, we did not detect increased Ti levels in the placenta or in fetal brain and liver tissues, suggesting that the majority of the maternally administered TiO_2_ particles did not accumulate in or cross the maternal-fetal interface. Therefore, it seems unlikely that a direct fetal exposure to TiO_2_ NPs is responsible for the subsequent emergence of behavioral abnormalities. Rather, the effects of prenatal TiO_2_ exposure may be driven by pathophysiological processes that arise in the maternal system and induce secondary effects in the offspring.

At present, the nature of these pathophysiological processes remains elusive and warrants identification in future studies. One candidate mechanism may involve alterations in the maternal immune system. In support of this hypothesis, it was previously shown that exposure to TiO_2_ NPs can induce signs of inflammation and other immune abnormalities, both in vivo and in vitro^58–60^. Given that abnormal immune functions and maternal inflammation have themselves been implicated in the etiology of ASD^7–14^, a link between prenatal exposure to TiO_2_, immune abnormalities, and emergence of ASD-related deficits seems biologically plausible. This hypothesis also warrants further exploration in view of the parallels between our findings and those reported in animal models of prenatal inflammation and other immune abnormalities. Indeed, prenatal exposure to immune challenges such as viral- or bacterial-like acute phase responses^7,61–63^ or allergy-like immune imbalances^11,64^ have been shown to induce similar ASD-related behavioral abnormalities as those reported here.

An alternative (but mutually not exclusive) possibility is that maternal TiO_2_ exposure could cause abnormal development and functions of the placenta even in the absence of placental accumulation of TiO_2_ NPs. The role of placental abnormalities in the etiology of ASD has received increasing recognition in recent years^15,65^. Being the critical sustenance delivery system for the fetus, placental health is critical for fetal growth and development^66^. Importantly, acting as the interface to communicate maternal nutritional and environmental statuses, the placenta rapidly responds to alterations in the maternal milieu and integrates the pathophysiological effects induced by a number of environmental adversities^66^. While various NPs have been associated with reproductive toxicity^67^, recent evidence suggests that this may also be the case for TiO_2_ NPs. For example, it was recently shown in mice that maternal exposure to TiO_2_ starting from conception to late gestation affected placental micronutrients and reduced placental weights^68^. Hence, the disruption of placental development and function may represent another possible mechanism by which maternal exposure to nano-sized TiO_2_ can induce ASD-relevant behavioral abnormalities in the offspring.

A third possible mechanism underlying the disruption of behavioral development following prenatal TiO_2_ exposure may involve changes in the maternal microbiota^69^. This hypothesis warrants exploration in view of the accumulating evidence suggesting that dysbiosis of the maternal microbiome can induce altered brain and behavioral development in the offspring with relevance to ASD and beyond^70^. In fact, alterations in the maternal microbiome may represent a common pathophysiological mechanism mediating the negative effects of a number of prenatal adversities, including maternal exposure to stress^71^, immune activation^72^, and high-fat diets^73^.

### Extrapolation to human exposures

The continuous rise in TiO_2_-containing products increases the human exposure to nano-sized TiO_2_. For example, the dietary intake of TiO_2_ in the US is estimated to be 1–2 mg/kg body weight per day for children, and 0.2–0.7 mg/kg body weight per day for other age groups^24,26^. Food-grade TiO_2_ particles are frequently used as white colorant and typically have a mean size of >100 nm, thus exceeding nano-size scales^24^. Nevertheless, approximately up to one third of TiO_2_ particles in common food products are nano-sized (<100 nm)^24,26^, suggesting that the daily ingestion of TiO_2_ NPs via common food products is considerable. According to recent bioavailability studies in humans, however, only a small proportion (~0.1%) of the ingested TiO_2_ NPs reaches the systemic circulation^74^. The low bioavailability of orally administered TiO_2_ NPs contrast the bioavailability of TiO_2_ NPs in our study, which was 100% as a result of the i.v. administration regimen. Given these differences in bioavailability, one could argue that our findings are artificial, and therefore, irrelevant for human conditions, where most of the TiO_2_ NPs reach the body via the oral route or inhalation^26,34,35,75^. While we appreciate the limitations of the chosen experimental design, we deem our study relevant for various reasons. First, given that TiO_2_ NPs can persist in tissues such as liver and spleen once absorbed^33^, it is likely that even limited systemic absorption can result in tissue accumulation, especially upon chronic exposure^26,33^. Secondly, our study emphasizes that a single exposure to high doses of TiO_2_ NPs in prenatal life is sufficient to induce long-term behavioral changes. Nano-sized TiO_2_ has been in discussion for the future use in biomedical applications, whereby the particles would serve as drug carriers and administered via the i.v. route^35,76^. Thus, together with other preclinical studies that were based on prenatal i.v. exposure to TiO_2_ NPs^33,37,56,57,77^, our findings emphasize the need of a careful assessment of the possible benefits and risks of administering nano-sized TiO_2_ to pregnant women.

### Limitations

Our study has a number of limitations. First, it did not aim at identifying the post-acute mechanisms mediating the effects of maternal TiO_2_ exposure on behavioral development in the offspring. Second, the characterization of the negative effects of prenatal TiO_2_ exposure was conducted exclusively at the behavioral level and did not encompass investigations of brain specimen. Hence, our study did not provide a link between TiO_2_-induced behavioral alterations and dysfunctions in specific neuronal and/or glial systems. Finally, even if we included a number of behavioral tests, the behavioral characterization of the effects of prenatal TiO_2_ exposure was far from exhaustive and did, for example, not involve the assessment of cognitive functions. With respect to the latter, future studies should also investigate whether this environmental hazard affects cognitive flexibility and working memory, both of which are altered in a subset of cases with ASD and related neurodevelopmental disorders^78,79^.

## Conclusion

Our study provides a first set of preliminary data suggesting that prenatal exposure to nano-sized TiO_2_ can induce behavioral deficits relevant to ASD and related neurodevelopmental disorders. Human epidemiological studies exploring associations between TiO_2_ exposure and health outcomes are still rare and largely confined to the research of cancer and pulmonary diseases^80–82^. Hence, our findings cannot be compared directly to human epidemiological data collected in the context of ASD, nor should they be taken to predict ASD risk in human populations. At the same time, however, our findings may provide an incentive for epidemiologists and basic scientists alike to further examine the role of prenatal TiO_2_ exposure in the etiology of ASD and other neurodevelopmental disorders. Such studies may also help to ascertain whether the increasing use of NPs such as nano-sized TiO_2_ contributes in some way to the rise in the incidence rates of these disorders.

## Electronic supplementary material

Supplementary Information
