# Supplementary material for: Prenatal exposure to TiO2 nanoparticles in mice causes behavioral deficits with relevance to autism spectrum disorder and beyond
Source: Transl Psychiatry. 2018 Sep 20;8:193. doi: 10.1038/s41398-018-0251-2 (PMC6148221; doi:10.1038/s41398-018-0251-2)
Supplement: Supplementary file 1 — Supplementary Information [file 41398_2018_251_MOESM1_ESM.docx]

**SUPPLEMENTARY INFORMATION**

**Prenatal exposure to TiO_2_ nanoparticles in mice causes behavioral deficits with relevance to autism spectrum disorder and beyond**

Tina Notter^1,2^, Leonie Aengenheister^3^, Ulrike Weber-Stadlbauer^1,2^, Hanspeter Naegeli^1^, Peter Wick^3^, Urs Meyer^1,2,§,^*, Tina Buerki-Thurnherr^3,§^

^1^Institute of Pharmacology and Toxicology, University of Zurich-Vetsuisse, Winterthurerstrasse 260, 8057 Zurich, Switzerland.

^2^Neuroscience Center Zurich, University of Zurich and ETH Zurich, Winterthurerstrasse 190, 8057 Zurich Zurich, Switzerland.

^3^Particles-Biology Interactions, Empa, Swiss Federal Laboratories for Materials Science and Technology, Lerchenfeldstrasse 5, 9014 St. Gallen, Switzerland.

^§^These authors share senior authorship.

**Corresponding author:*

Prof. Urs Meyer, Institute of Pharmacology and Toxicology, University of Zurich-Vetsuisse, Winterthurerstrasse 260, 8057 Zurich, Switzerland.

Tel: +41 44 635 88 44, E-mail: urs.meyer@vetpharm.uzh.ch

Author e-mail addresses:

Tina Notter: [tina.notter@uzh.ch](mailto:tina.notter@uzh.ch)

Leonie Aengenheister: [leonie.aengenheister@empa.ch](mailto:email:%20leonie.aengenheister@empa.ch)

Ulrike Weber-Stadlbauer: [ulrike.weber@uzh.ch](mailto:ulrike.weber@uzh.ch)

Hanspeter Naegeli: [hanspeter.naegeli@vetpharm.uzh.ch](mailto:hanspeter.naegeli@vetpharm.uzh.ch)

Peter Wick: [peter.wick@empa.ch](mailto:peter.wick@empa.ch)

Urs Meyer: [urs.meyer@vetpharm.uzh.ch](mailto:urs.meyer@vetpharm.uzh.ch)

Tina Buerki-Thurnherr: [tina.buerki@empa.ch](mailto:tina.buerki@empa.ch)

**Supplementary Material and Methods**

**Animals**

C57Bl6/N mice were used throughout this study. Female and male breeders were obtained from Charles River Laboratories (Sulzfeld, Germany) and kept in our in-house, specific-pathogen-free facility. All animal breeding and holding rooms were temperature- and humidity-controlled (21 ± 1 °C, 55 ± 5%) and kept under a reversed light–dark cycle (lights off: 8:00 A.M. to 8:00 P.M.). All animals had *ad libitum* access to food (Kliba 3436, Kaiseraugst, Switzerland) and water. All procedures involving animal experimentation had been previously approved by the Cantonal Veterinarian’s Office of Zurich, and all efforts were made to minimize the number of animals used and their suffering.

**Characterization of TiO_2_ NP suspension**

TiO_2_ anatase NPs (NM-101) were provided by the Joint Research Centre (JRC, Ispra, Italy). The TiO_2_ NP powder was dissolved in sterile and pyrogen-free phosphate-buffered saline (PBS) (D8537, Sigma-Aldrich, Switzerland) (5 mg/mL) via probe sonication (5 min at 13 W, on ice; Branson sonifier 250, Branson Ultrasonic Co., Danbury, CT, USA). The dispersion was further diluted (1:10) with sterile and pyrogen-free PBS to yield the lower dose (0.5 mg/mL) and was characterized using transmission electron microscopy (TEM) as described elsewhere^1^. **Supplementary Table 2** provides a summary of the characterization data supplied by the Joint Research Centre ^1^ and experimental data obtained after dispersion in PBS solution. The zeta potential was measured in a 0.5 mg/mL TiO_2_ NP suspension at 25 °C via dynamic light scattering (DLS) using a Zetasizer Nano-ZS90 (Malvern Instruments, Worcestershire, UK). The hydrodynamic diameter was determined in a 100 µg/mL TiO_2_ NP suspension by Nanoparticle Tracking Analysis (NTA 3.1 Build 3.1.54; Nanosight NS500, Malvern, Worcestershire, UK).

**Prenatal TiO_2_ NP exposure**

C57BL6/N female mice were subjected to a timed mating procedure as described previously ^2^. Pregnant dams were subjected to a single injection of the 100 µg or 1000 µg TiO_2_ NP solution (see above) or vehicle solution (PBS) on gestation day 9 (GD) 9. A volume of 200 µL of either 5 mg/mL TiO_2_ NP suspension (= 1000 µg dose), 0.5 mg/mL TiO_2_ NP suspension (= 100 µg dose) or PBS was administered intravenously (i.v) into the tail vein under mild physical constrain as described previously ^2^.

The two doses were selected based on previous studies in rodents, which investigated the effects of maternal TiO_2_ NP exposure on fetal development ^3-6^. As a first proof-of-principle study, we chose to administer TiO_2_ NP using the i.v. route as it allows a precise control of dosing and gestational timing. The gestational window of maternal TiO_2_ exposure (i.e., GD 9) corresponds roughly to human gestational weeks 4 to 5 in terms of limbic neurogenesis ^7^. It was selected based on human epidemiological studies suggesting that the first trimester of human pregnancy is particularly sensitive in the context of environmental adversities and neurodevelopmental disorders such as ASD ^8-10^.

**Experimental groups and allocation of offspring**

Two cohorts of pregnant mice were generated under identical experimental and housing conditions (see above). The first cohort of dams was designated to fetal developmental studies, in which the dams were killed 1 week after treatment with TiO_2_ NP or vehicle. In this cohort, 7 dams were injected with PBS (0 µg), 10 dams were injected with 100 µg of TiO_2_ NP, and 8 dams were injected with 1000 µg of TiO_2_ NP. Only 1 fetus per dam was randomly assigned to the developmental studies in order to minimize litter effects ^11^. Hence, the number of fetuses was equal to the number of treated dams in each experimental group. The second cohort of dams was used to generate offspring for behavioral studies. To this end, 8 dams were injected with PBS (0 µg), 8 dams were injected with 100 µg of TiO_2_ NP, and 9 dams were injected with 1000 µg of TiO_2_ NP. Whenever possible, only 1 male and 1 female offspring per litter were randomly selected for the subsequent behavioral investigations to minimize potential confounds arising from litter effects (see **Supplementary Table 1**) ^11^. This led to a group size of *N*(0 µg) = 16 (8m, 8f), *N*(100 µg) = 16 (8 m, 8 f), and *N*(1000 µg) = 17 (9m, 8f) in the ultrasonic vocalization (USV) test (see below), which was conducted in neonatal offspring (see **Supplementary Table 1**). As described in more detail below, the neonatal USV test requires a temporary separation form the rearing mother and littermates, which in turn may represent a neonatal stressor influencing brain development per se ^12^. Therefore, all other behavioral tests (see below) were conducted in the remaining offspring that were not subjected to neonatal USV testing. The group size of these remaining offspring was *N* (0 µg) = 20 (10m, 10f), *N*(100 µg) = 19 (10m, 9f), and *N*(1000 µg) = 20 (10m, 10f). They were weaned and sexed on postnatal day (PND) 21. Littermates of the same sex were caged separately and maintained in groups of 3 to 4 animals per cage. They were kept in temperature- and humidity-controlled holding rooms under a reversed light–dark cycle and *ad libitum* food access as described above. Behavioral testing commenced one week after weaning (i.e., on PND 28) and was completed on PND 42. Hence, testing was conducted during the juvenile period in order to account for the typical juvenile onset of ASD ^13, 14^.

Supplementary Table 1: Summary of animals used for and order of behavioral testing.

| **Behavioral Test** | **0 µg TiO_2_** | **100 µg TiO_2_** | **1000 µg TiO_2_** |
| --- | --- | --- | --- |
|  | # Females, males (dams) | # Females, males (dams) | # Females, males (dams) |
| USV test | 8, 8 (8) | 8, 8 (8) | 8, 9 (9) |
| 1. Open field test | 10,10 (8) | 9, 10 (8) | 10,10 (9) |
| 2. Social interaction test | 10,10 (8) | 9, 10 (8) | 10,10 (9) |
| 3. Self-grooming test | 10,10 (8) | 9, 10 (8) | 10,10 (9) |
| 4. Prepulse inhibition test | 10,10 (8) | 9, 10 (8) | 10,10 (9) |

**Maternal and fetal tissue collection for ICP-MS and evaluation of fetal development**

Rutile-type TiO_2_ NP exposure in mid-to-late gestation was previously shown to induce fetal malformations ^6^. To assess whether a single TiO_2_ NP exposure in early gestation (GD 9) similarly affects fetal outcomes, we assessed gross characteristics of fetal development. In addition, we aimed at determining the distribution of TiO_2_ NPs in maternal and fetal tissues. For these purposes, pregnant mice were killed by decapitation 1 week after treatment with TiO_2_ NPs or vehicle (i.e., on GD 16). Trunk blood was collected in heparinized tubes (Microvette CB 300 LH, Sarstedt, Nümbrecht, Germany) for sector field-inductively coupled plasma mass spectrometry (SF-ICP-MS) measurements of Ti contents (see below) in maternal plasma. Plasma was separated by centrifugation (2000 × g, 5 min) and stored at −20 °C until analyses. Maternal livers and spleens were frozen with powdered dry ice immediately after collection and stored at -80°C until SF-ICP-MS analyses were performed (see below).

The dams’ abdominal cavities were exposed, and the uteri were removed and placed in a petri dish filled with ice-cold PBS. The uteri were then dissected to collect entire fetuses and placental tissues. The latter were immediately frozen with powdered dry ice and stored at -80°C for later SF-ICP-MS analyses (see below). Fetuses were weighed using an analytical balance immediately after dissection (Mettler-Toledo AE 240, Switzerland), and either immersion-fixed in 4% phosphate-buffered paraformaldehyde (PFA) for 24 hours (1 fetus per dam) or further processed to collect fetal brain and liver tissues as previously described ^15^. Fetal length was assessed in fixed fetuses by measuring the distance between nose bone and tail root. Fetal brain weight was measured using and analytical balance immediately after dissection. Fetal brain and liver tissues stemming from 1 fetus per dam were frozen with powdered dry ice and stored at -80°C for later SF-ICP-MS analyses (see below). These assessments were carried out by an experimenter who was blind to the treatment groups.

**Quantification of Ti tissue contents using SF-ICP-MS**

Using the first cohort of dams (see above), 5 dams per group were randomly selected to quantify the contents of Ti in fetal and maternal tissues. Maternal spleen, maternal liver, maternal plasma, placenta, fetal brain, and fetal liver were weighed and were digested in 2 mL concentrated nitric acid (67%, NORMATOM, VWR Chemicals, Vienna, Austria) and 1 mL ultrapure water using a microwave (turboWAVE Inert, MLS GmbH, Leutkirch, Germany). Digested samples were further diluted using ultrapure water. Ti content was determined by SF-ICP-MS (Element 2, Thermo Finnigan, Bremen, Germany) with external calibration ranging from 0.5 – 100 µg/L. Isotopes ^47^Ti, ^48^Ti and ^49^Ti were analyzed in low and medium resolution to identify potential interferences. ^47^Ti determined in the medium resolution was used for quantification. The associated limit of detection (LOD) defined as the mean of 5 blank measurements plus 3 times its standard deviation was 0.497 µg/L.

**Neonatal ultrasonic vocalization test**

Ultrasonic vocalization (USV) is a form of vocal communication in mice. When separated from their rearing mothers and/or littermates, neonatal mice emit USVs that can be measured to assess the communication between pups and their mothers ^16, 17^. Deficits in this early form of pub-to-mother communication have been implicated in neurodevelopmental disorders such as ASD and represent core behavioral deficits in ASD-relevant mouse models ^18-20^.

On PND 6, two designated pups (1 female and 1 male) per litter were removed from their home cage and placed in a 15 × 15 cm white Plexiglas box located inside a sound-attenuating chamber with an ambient temperature of 23.5-23.6°C. The chambers were always assigned to either male or female animals. USVs were recorded and analyzed using the UltraVox XT version 3.0 software (Noldus Information Technology, Netherland). They were recorded for 5 min using full-sound spectrum digital microphones, which were provided with the UltraVox XT3 package. The microphones were connected via a USB connector (UltraVox XT version 3.0) to a computer to record the USVs. Vocalizations were recorded using a sampling rate of 250 kHz. After 5 min of recording, the pups were removed from the Plexiglas box and killed by decapitation.

All acoustic signals were measured and analyzed by an automated call detection system that was provided with the UltraVox XT3 software. A short-time Fourier transformation (SFT) was applied to the recorded data (265 SFT-length, 50% time window overlap), after which individual USVs were identified using a threshold-based algorithm provided by the UltraVox XT. According to previous studies ^21^, the algorithm was set to detect USVs lasting more than 10 ms each and a cut-off frequency of 30 kHz. The detection accuracy was additionally verified by an experienced investigator who was blind to the treatment conditions. The total number of emitted calls, mean duration of calls, and mean dominant frequency (i.e., the average value of frequency at maximum amplitude) were analyzed.

**Social interaction test**

Social behavior is commonly referred to behavior that takes place in a social context and results from the interaction between individuals (of the same species). As mice (like most other rodents) are highly social animals, social interaction can be efficiently studied under experimental conditions ^20, 22^. Importantly, evaluation of social interaction is of particular relevance to ASD as it represents one of the cardinal behavioral deficits characterizing this disorder ^20, 22^.

The social interaction test was performed using methods established and validated before ^23-25^. In this test, sociability was indexed as the relative exploration time between an unfamiliar, congenic mouse of the same sex and an inanimate dummy object.^23-25^ The apparatus consisted of three identical arms (50 × 9 cm; length × width) surrounded by 10 cm high, opaque Plexiglas walls. The three arms radiated from a central triangle (8 cm on each side) and spaced 120° from each other. Two of the three arms contained rectangular wire grid cages (13 × 8 × 10 cm, length × width × height; bars horizontally and vertically spaced 9 mm apart). The third arm did not contain a metal wire cage and served as the start zone (‘start arm’ see below). On the first day, all animals were habituated to the apparatus by being allowed to explore the Y-maze for 5 min. This served to familiarize the test animals with the apparatus and to reduce novelty related locomotor hyperactivity that may potentially confound social interaction during the critical test phase. The following day animals were subjected to the social interaction test.

During the test day, one metal wire cage contained an unfamiliar, congenic mouse of the same sex (4-6 weeks of age), whereas the other wire cage contained an inanimate dummy object. The latter was made of black LEGO™ bricks and took the shape of a mouse. The allocation of the unfamiliar live mouse and inanimate dummy object to the two wire cages was counterbalanced across experimental groups. To start a test trial, the test mouse was gently placed in the start arm and allowed to explore freely for 5 min. Behavioral observations were made by an experimenter who was blind to the experimental conditions, and social interaction was defined as nose contact within a 2-cm interaction zone. The relative time spent with the live mouse was calculated by the formula ([time spent with the mouse]/[time spent with the inanimate object + time spent with the mouse]) × 100 and used to compare the relative exploration time between the unfamiliar mouse and the inanimate dummy object. The total distance moved during the test was also measured to analyze general exploratory activity. This was achieved by a digital camera mounted above the apparatus, which provided images at a rate of 5 Hz that were transmitted to a PC running the EthoVision tracking system (Noldus Information Technology, The Netherlands).

**Self-grooming test**

Atypical repetitive/stereotyped behavior represents another cardinal symptom of ASD ^26^. In order to measure repetitive/stereotyped behaviors, the animals were subjected to a self-grooming test. Self-grooming is an innate and evolutionary conserved behavior that follows a conserved pattern of sequential, self-directed behaviors, and as such, it is widely used to assess stereotyped/repetitive behaviors in preclinical ASD research ^27^. The self-grooming test followed procedures described by Malkova et al. ^19^ with small modifications. In brief, the animals were placed in a clear glass beaker (10 cm diameter × 18 cm tall) that was located in a chamber of the Multi Conditioning System (TSE Systems GmbH, Bad-Homburg, Germany). After a 10-min habituation phase in the beaker, the animals were video-recorded for a further 10 min. An investigator blinded to the experimental conditions analyzed the videos for self-grooming, which was indexed as the cumulative time spent grooming. All testing beakers were cleaned with water between tests to remove olfactory cues.

**Prepulse inhibition test**

Sensorimotor gating was assessed using the paradigm of prepulse inhibition (PPI) of the acoustic startle reflex. PPI of the acoustic startle reflex refers to the reduction in startle reaction in response to a startle-eliciting pulse stimulus when it is shortly preceded by a weak prepulse stimulus ^2^. The rationale for selecting this test was based on the reports of altered PPI in ASD children ^28^.

The apparatus consisted of four startle chambers for mice (San Diego Instruments, San Diego, CA, USA) and has been fully described elsewhere ^2^. In the demonstration of PPI, the animals were presented with a series of discrete trials comprising a mixture of 4 trial types. These included pulse-alone trials, prepulse-plus-pulse trials, prepulse-alone trials, and no-stimulus trials in which no discrete stimulus other than the constant background noise was presented. The pulse and prepulse stimuli used were in the form of a sudden elevation in broadband white noise level (sustaining for 40 and 20 ms, respectively) from the background (65 dB_A_), with a rise time of 0.2-1.0 ms. In all trials, 3 different intensities of pulse (100, 110, and 120 dB_A_) and 3 intensities of prepulse (71, 77, and 83 dB_A_, which corresponded to +6, +12, and +18 dB_A_ above background, respectively) were applied. The stimulus-onset asynchrony of the prepulse and pulse stimuli on all prepulse-plus-pulse trials was 100 ms (onset-to-onset).

The protocol used for the PPI test was extensively validated before ^23, 25, 29^. A session began with the animals being placed into the Plexiglas enclosure. They were acclimatized to the apparatus for 2 min before the first trial began. The first 6 trials consisted of 6 startle-alone trials; such trials served to habituate and stabilize the animals’ startle response and were not included in the analysis. Subsequently, the animals were presented with 10 blocks of discrete test trials. Each block consisted of the following: three pulse-alone trials (100, 110, or 120 dB_A_), 3 prepulse-alone trials (+6, +12, or +18 dB_A_ above background), 9 possible combinations of prepulse-plus-pulse trials (3 levels of pulse × 3 levels of prepulse), and one no stimulus trial. The 16 discrete trials within each block were presented in a pseudorandom order, with a variable interval of 15 s on average (ranging from 10 to 20 s). For each of the 3 pulse intensities (100, 110, or 120 dB_A_), PPI was indexed by percent inhibition of the startle response obtained in the pulse-alone trials by the following expression: 100% × [1 − (mean reactivity on prepulse-plus-pulse trials/mean reactivity on pulse-alone trials)], for each animal, and at each of the three possible prepulse intensities (+6, +12, or +18 dB_A_ above background). Reactivity to pulse-alone trials and prepulse-alone trials were also analyzed in order to evaluate startle responses and prepulse-induced reactivity.

**Open-field test**

Innate anxiety was assessed using a standard open-field exploration task ^30^. The apparatus consisted of 4 identical open-field arenas (40 × 40 × 35-cm high) made of opaque acryl glass as described before ^31^. They were located in a testing room under dim lighting (approximately 35 lux as measured in the center of the arenas). For the purpose of data collection, the arena was conceptually partitioned into a centre zone (measuring 13.5 × 13.5 cm^2^) in the middle of the area. A digital camera was mounted directly above the four arenas. Images were captured at a rate of 5 Hz and transmitted to a PC running the EthoVision tracking system (Noldus Information Technology, Wageningen, The Netherlands). Mice were gently placed into the open-field and were allowed to freely explore the arena for 10 min. Innate anxiety was indexed by the time spent in the center zone during 10 min of free exploration.

**Statistical analyses**

All data were analyzed using parametric analysis of variance (ANOVA). Tissue contents of Ti measured by SF-ICP-MS were analyzed using a 3 × 6 (prenatal treatment × tissue compartment) ANOVA. Fetal weights, fetal brain weights, fetal length, and litter sizes were analyzed using one-way ANOVA. The male/female ratio of delivered offspring was analyzed using a 3 × 2 (prenatal treatment × sex) ANOVA, whereas offspring body weights were analyzed using a 3 × 2 × 3 (prenatal treatment × sex × age) ANOVA. Behavioral data from the USV, open field, social interaction, and self-grooming tests were analyzed using 3 × 2 (prenatal treatment × sex) ANOVAs. Percent PPI was analyzed using a 3 × 2 × 3 × 3 (prenatal treatment × sex × prepulse level × pulse level) ANOVA, and reactivity to pulse-alone trials and prepulse alone trials were analyzed using 3 × 2 × 3 (prenatal treatment × sex × pulse level) and 3 × 2 × 3 (prenatal treatment × sex × prepulse level) ANOVAs, respectively. Whenever appropriate, all ANOVAs were followed by Fisher’s least significant difference (LSD) post-hoc tests. All statistical analyses were performed using StatView (version 5.0; Abacus, Phoenix, AZ, USA) implemented on a PC running the Windows XP operating system, and Prism software (version 7.0; GraphPad Software, La Jolla, CA, USA). Statistical significance was set at *p* < 0.05 for all tests.

**Supplementary Results**

**Characterization of TiO_2_ NPs**

TEM analysis showed that TiO_2_ suspensions in water mostly consisted of aggregates and agglomerates (size range of 10-170 nm) containing primary particles with an approximate size of 5-6 nm ^1^. The amount of aggregates/agglomerates smaller than 100 nm, 50 nm and 10 nm was 95.2 %, 77.3% and 10.7%, respectively. NTA demonstrated that the hydrodynamic diameter of the TiO_2_ material in PBS vehicle was 89.0 ± 54.1 nm (**Supplementary Table 1**). DLS analysis showed that the zeta potential of the TiO_2_ NPs in PBS was -21.1 ± 1.2 mV (**Supplementary Table 1**). Hence, the NP characterization indicated that the TiO_2_ suspension used here contains particles that fall into the category of nano-sized materials ^32^.

Supplementary Table 2: Characterization of TiO_2_ NPs (NM-101)

|  | **TiO_2_ (NM-101)** |
| --- | --- |
| TEM: Primary particle size [nm]^a^ | 5 - 6 |
| TEM: Particle size distribution [nm] ^a^ | <100 → 95.2 %  <50 → 77.3 %  <10 → 10.7 % |
| SAXS/BET: Specific surface area [m^2^/g] ^a^ | 170/316 |
| XRD: Crystalline phase ^a^ | Anatase |
| Zeta potential in PBS [mV] ^b^ | -21.1 ± 1.2 |
| Hydrodynamic diameter in PBS [nm] ^b^ | 89.0 ± 54.1 |

Abbreviations: TEM: transmission electron microscopy; SAXS: Small angle X-ray scattering; BET: Brunauer Emmett Teller method; XRD: X-ray diffraction. ^a^Information provided by JRC ^1^. ^b^Experimentally determined parameters: Zeta potential represents mean ± SD; Hydrodynamic diameter represents mode ± SD.


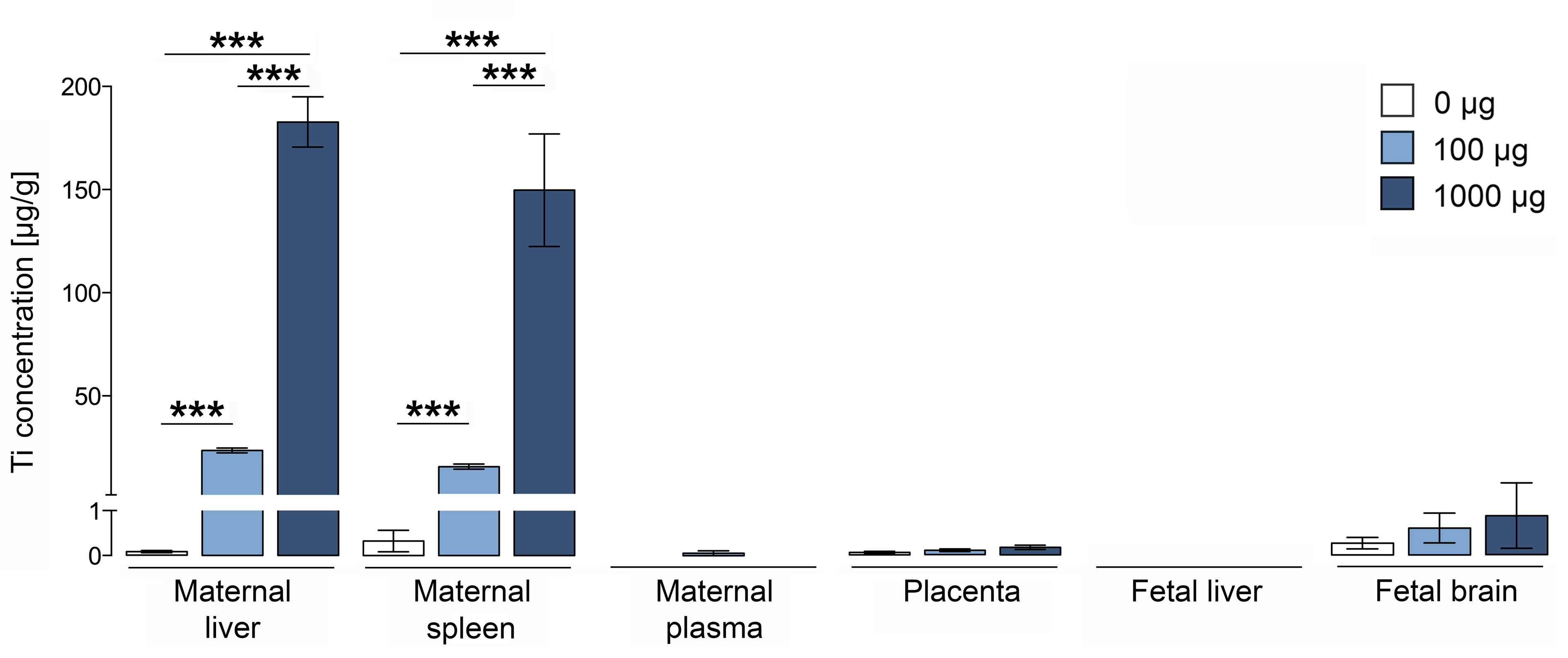


**Supplementary Fig. 1.** Ti contents in maternal and fetal tissues 7 days after administration of 100 or 1000 µg TiO_2_ nanoparticles or vehicle (0 µg) on gestation day 9. The bar plot depicts the content of Ti (µg/g tissue) measured using SF-ICP-MS in the different maternal and fetal tissues. ****p* < 0.001; *N*(0 µg) = 5, *N*(100 µg) = 5, *N*(1000 µg) = 5. All values are means ± SEM.

**Supplementary Fig. 2.** The effect of prenatal TiO2 NP exposure on fetal development, pregnancy outcomes and postnatal development. (a) The photographs depict representative fetuses collected 7 days after maternal administration of 100 or 1000 µg TiO2 nanoparticles or vehicle (0 µg) on gestation day 9. Scale bar 1 cm. (b) The bar plots depict fetal length (cm), fetal weights (mg), and fetal brain weights (mg). N(0 µg) = 7, N(100 µg) = 10, N(1000 µg) = 8. (c) The bar plot shows the number of delivered pups per dam. N(0 µg) = 8, N(100 µg) = 8, N(1000 µg) = 9. (d) The bar plot depicts the percentage of male (grey) and female (white) offspring per litter (male/female ratio). N(0 µg) = 8, N(100 µg) = 8, N(1000 µg) = 9. (e) The bar plots illustrate the body weights (g) of the offspring on postnatal day (PND) 6, 21 and 28. ***p < 0.001, reflecting the significant age differences regardless of treatment; PND 6: N(0 µg) = 16 (8m, 8f), N(100 µg) = 16 (8 m, 8 f), N(1000 µg) = 17 (9m, 8f). PND 21 and 28: N(0 µg) = 20 (10m, 10f), N(100 µg) = 19 (10m, 9f), and N(1000 µg) = 20 (10m, 10f). All values are means ± SEM.

**Supplementary References**

1. Report J. JRC-Nanomaterials-Repository: 2014: . [*http://ihcpjrceceuropaeu/our_activities/nanotechnology/nanomaterials-repository*](http://ihcpjrceceuropaeu/our_activities/nanotechnology/nanomaterials-repository) 2014 **Accessed 12 July 2017**.

2. Meyer U, Feldon J, Schedlowski M, Yee BK. Towards an immuno-precipitated neurodevelopmental animal model of schizophrenia. *Neuroscience and biobehavioral reviews* 2005; **29**(6)**:** 913-947.

3. Mohammadipour A, Fazel A, Haghir H, Motejaded F, Rafatpanah H, Zabihi H*, et al*. Maternal exposure to titanium dioxide nanoparticles during pregnancy; impaired memory and decreased hippocampal cell proliferation in rat offspring. *Environmental toxicology and pharmacology* 2014; **37**(2)**:** 617-625.

4. Takeda K, Suzuki K, Ishihara A, Kubo-Irie M, Fujimoto R, Tabata M*, et al*. Nanoparticles transferred from pregnant mice to their offspring can damage the genital and cranial nerve systems. *Journal of Health Science* 2009; **55**(1)**:** 95-102.

5. Takahashi Y, Mizuo K, Shinkai Y, Oshio S, Takeda K. Prenatal exposure to titanium dioxide nanoparticles increases dopamine levels in the prefrontal cortex and neostriatum of mice. *The Journal of toxicological sciences* 2010; **35**(5)**:** 749-756.

6. Yamashita K, Yoshioka Y, Higashisaka K, Mimura K, Morishita Y, Nozaki M*, et al*. Silica and titanium dioxide nanoparticles cause pregnancy complications in mice. *Nature nanotechnology* 2011; **6**(5)**:** 321-328.

7. Brains TTADM. <http://translatingtime.net/translate>. **Accessed 15 December 2017**.

8. Meyer U, Yee BK, Feldon J. The neurodevelopmental impact of prenatal infections at different times of pregnancy: the earlier the worse? *The Neuroscientist : a review journal bringing neurobiology, neurology and psychiatry* 2007; **13**(3)**:** 241-256.

9. Atladottir HO, Thorsen P, Ostergaard L, Schendel DE, Lemcke S, Abdallah M*, et al*. Maternal infection requiring hospitalization during pregnancy and autism spectrum disorders. *Journal of autism and developmental disorders* 2010; **40**(12)**:** 1423-1430.

10. Jiang HY, Xu LL, Shao L, Xia RM, Yu ZH, Ling ZX*, et al*. Maternal infection during pregnancy and risk of autism spectrum disorders: A systematic review and meta-analysis. *Brain, behavior, and immunity* 2016; **58:** 165-172.

11. Zorrilla EP. Multiparous species present problems (and possibilities) to developmentalists. *Developmental psychobiology* 1997; **30**(2)**:** 141-150.

12. Lehmann J, Feldon J. Long-term biobehavioral effects of maternal separation in the rat: consistent or confusing? *Reviews in the neurosciences* 2000; **11**(4)**:** 383-408.

13. Landa RJ, Holman KC, Garrett-Mayer E. Social and communication development in toddlers with early and later diagnosis of autism spectrum disorders. *Archives of general psychiatry* 2007; **64**(7)**:** 853-864.

14. Rapin I, Tuchman RF. Autism: definition, neurobiology, screening, diagnosis. *Pediatric clinics of North America* 2008; **55**(5)**:** 1129-1146, viii.

15. Meyer U, Nyffeler M, Engler A, Urwyler A, Schedlowski M, Knuesel I*, et al*. The time of prenatal immune challenge determines the specificity of inflammation-mediated brain and behavioral pathology. *The Journal of neuroscience : the official journal of the Society for Neuroscience* 2006; **26**(18)**:** 4752-4762.

16. Hofer MA, Shair HN, Brunelli SA. Ultrasonic vocalizations in rat and mouse pups. *Current protocols in neuroscience* 2002; **Chapter 8:** Unit 8.14.

17. Scattoni ML, Crawley J, Ricceri L. Ultrasonic vocalizations: a tool for behavioural phenotyping of mouse models of neurodevelopmental disorders. *Neuroscience and biobehavioral reviews* 2009; **33**(4)**:** 508-515.

18. Mosienko V, Beis D, Alenina N, Wohr M. Reduced isolation-induced pup ultrasonic communication in mouse pups lacking brain serotonin. *Molecular autism* 2015; **6:** 13.

19. Malkova NV, Yu CZ, Hsiao EY, Moore MJ, Patterson PH. Maternal immune activation yields offspring displaying mouse versions of the three core symptoms of autism. *Brain, behavior, and immunity* 2012; **26**(4)**:** 607-616.

20. Silverman JL, Yang M, Lord C, Crawley JN. Behavioural phenotyping assays for mouse models of autism. *Nature reviews Neuroscience* 2010; **11**(7)**:** 490-502.

21. Braunschweig D, Golub MS, Koenig CM, Qi L, Pessah IN, Van de Water J*, et al*. Maternal autism-associated IgG antibodies delay development and produce anxiety in a mouse gestational transfer model. *Journal of neuroimmunology* 2012; **252**(1-2)**:** 56-65.

22. Crawley JN. Mouse behavioral assays relevant to the symptoms of autism. *Brain pathology (Zurich, Switzerland)* 2007; **17**(4)**:** 448-459.

23. Weber-Stadlbauer U, Richetto J, Labouesse MA, Bohacek J, Mansuy IM, Meyer U. Transgenerational transmission and modification of pathological traits induced by prenatal immune activation. *Molecular psychiatry* 2017; **22**(1)**:** 102-112.

24. Bitanihirwe BK, Peleg-Raibstein D, Mouttet F, Feldon J, Meyer U. Late prenatal immune activation in mice leads to behavioral and neurochemical abnormalities relevant to the negative symptoms of schizophrenia. *Neuropsychopharmacology : official publication of the American College of Neuropsychopharmacology* 2010; **35**(12)**:** 2462-2478.

25. Notter T, Coughlin JM, Gschwind T, Weber-Stadlbauer U, Wang Y, Kassiou M*, et al*. Translational evaluation of translocator protein as a marker of neuroinflammation in schizophrenia. *Molecular psychiatry* 2017.

26. Pasciuto E, Borrie SC, Kanellopoulos AK, Santos AR, Cappuyns E, D'Andrea L*, et al*. Autism Spectrum Disorders: Translating human deficits into mouse behavior. *Neurobiology of learning and memory* 2015; **124:** 71-87.

27. Kalueff AV, Stewart AM, Song C, Berridge KC, Graybiel AM, Fentress JC. Neurobiology of rodent self-grooming and its value for translational neuroscience. *Nature reviews Neuroscience* 2016; **17**(1)**:** 45-59.

28. Madsen GF, Bilenberg N, Cantio C, Oranje B. Increased prepulse inhibition and sensitization of the startle reflex in autistic children. *Autism research : official journal of the International Society for Autism Research* 2014; **7**(1)**:** 94-103.

29. Vuillermot S, Weber L, Feldon J, Meyer U. A longitudinal examination of the neurodevelopmental impact of prenatal immune activation in mice reveals primary defects in dopaminergic development relevant to schizophrenia. *The Journal of neuroscience : the official journal of the Society for Neuroscience* 2010; **30**(4)**:** 1270-1287.

30. Belzung C, Griebel G. Measuring normal and pathological anxiety-like behaviour in mice: a review. *Behavioural brain research* 2001; **125**(1-2)**:** 141-149.

31. Stadlbauer U, Weber E, Langhans W, Meyer U. The Y2 receptor agonist PYY(3-36) increases the behavioural response to novelty and acute dopaminergic drug challenge in mice. *The international journal of neuropsychopharmacology* 2014; **17**(3)**:** 407-419.

32. Environment EC. Definition of a nanomaterial. [*http://eceuropaeu/environment/chemicals/nanotech/faq/definition_enhtm*](http://eceuropaeu/environment/chemicals/nanotech/faq/definition_enhtm) **Accessed 8 January 2018**.
